# Creating groups with similar expected behavioural response in randomized controlled trials: a fuzzy cognitive map approach

**DOI:** 10.1186/1471-2288-14-130

**Published:** 2014-12-12

**Authors:** Philippe J Giabbanelli, Rik Crutzen

**Affiliations:** Interdisciplinary Research in the Mathematical and Computational Sciences (IRMACS) Centre, Simon Fraser University, Burnaby, Canada; UKCRC Centre for Diet and Activity Research, MRC Epidemiology Unit, University of Cambridge School of Clinical Medicine, Box 285 Institute of Metabolic Science, Cambridge Biomedical Campus, Cambridge, CB2 0QQ UK; Department of Health Promotion, Maastricht University/CAPHRI, Maastricht, The Netherlands

**Keywords:** Allocation method, Artificial intelligence, Computational model, Randomization

## Abstract

**Background:**

Controlling bias is key to successful randomized controlled trials for behaviour change. Bias can be generated at multiple points during a study, for example, when participants are allocated to different groups. Several methods of allocations exist to randomly distribute participants over the groups such that their prognostic factors (e.g., socio-demographic variables) are similar, in an effort to keep participants’ outcomes comparable at baseline. Since it is challenging to create such groups when all prognostic factors are taken together, these factors are often balanced in isolation or only the ones deemed most relevant are balanced. However, the complex interactions among prognostic factors may lead to a poor estimate of behaviour, causing unbalanced groups at baseline, which may introduce accidental bias.

**Methods:**

We present a novel computational approach for allocating participants to different groups. Our approach automatically uses participants’ experiences to model (the interactions among) their prognostic factors and infer how their behaviour is expected to change under a given intervention. Participants are then allocated based on their inferred behaviour rather than on selected prognostic factors.

**Results:**

In order to assess the potential of our approach, we collected two datasets regarding the behaviour of participants (n = 430 and n = 187). The potential of the approach on larger sample sizes was examined using synthetic data. All three datasets highlighted that our approach could lead to groups with similar expected behavioural changes.

**Conclusions:**

The computational approach proposed here can complement existing statistical approaches when behaviours involve numerous complex relationships, and quantitative data is not readily available to model these relationships. The software implementing our approach and commonly used alternatives is provided at no charge to assist practitioners in the design of their own studies and to compare participants' allocations.

## Background

Randomized Controlled Trials (RCTs) are a powerful approach to conduct quantitative and comparative controlled experiments. However, many sources of bias can negatively affect the quality of the experiments. While carefully controlling bias is always required, the difficulty of doing so varies depending on the area. For example, RCTs for health behaviour change are more prone to bias than pharmaceutical trials, and reducing bias may also be more difficult for the former than latter condition [1, 2]. A statistical introduction to sources of bias can be found in Matthews [3], while an overview for a broader audience is provided in Jadad and Enkin [4].

**Figure 1:**
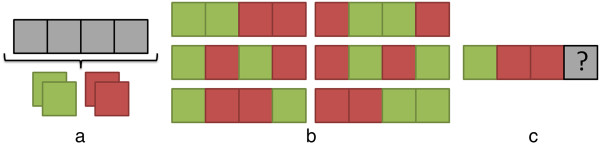
**Allocation in blocks.** An approach to allocating participants to two groups (red, green) is to use fixed blocks of size 4, which contain an equal number of participants from both groups **(a)**. This leads to six possible sequences of allocations **(b)**. However, if the three participants’ allocation is known, then it is possible to know the allocation of the fourth **(c)**.

A key characteristic of RCTs is that participants are randomly assigned to a group, which could be done by flipping a coin (for two groups) or throwing a dice (for more). However, this purely random technique can result in significant differences in sizes of treatment groups. This is is not an issue for bias in trial results, since the conventional analysis of randomised trials takes into account the fact that groups can be unbalanced [5]. Aiming at equal allocation ratio is nonetheless common practice (e.g. procedures in [6, 7]). Comparable groups sizes can be ensured by assigning patients in blocks (*random permuted blocks*). However, if blocks are of fixed size and contain a known distribution across treatment groups (Figure 1a-b), then it becomes possible to correctly guess how the last participants in a block will be allocated (Figure 1c). As blinding can be particularly difficult in some trials [1], the staff may treat a participant differently if the allocation was known, which introduces a *selection* bias. This illustrates the difficulties of creating a scheme that guarantees comparable groups while making it difficult for the staff to predict the group to which a participant will be assigned. The situation is even more challenging when considering that the balance among groups is not only a matter of having an equal number of participants, but that participants should also be balanced with respect to prognostic factors (e.g., socio-demographic or socio-cognitive variables related to the behavioural outcome). Indeed, the randomization scheme must achieve balance on confounding prognostic factors in order to avoid what is known as the *accidental* bias.

Two techniques that take into account prognostic factors are *stratification* and *minimization*. The former applies randomization for every *combination* of participant prognostics (e.g., to guarantee the same number of patients across groups for each age category *and* ethnicity), which may not be feasible in small trials and/or when the number of such combinations is very large. The latter handles this problem by ensuring a balance only in the *individual* prognostics as participants enter the trial (e.g., to guarantee the same number of patients across groups for each age category *or* ethnicity). Both techniques assume that the mechanisms by which the prognostics contribute to the trial outcome are unknown, thus they aim at controlling the distributions of prognostic factors and expect this to carry onto the distribution of outcomes. In this paper, we take a different approach to the allocation phase of RCTs for behaviour change by focusing on the distribution of outcomes. As will be illustrated throughout the paper, the advantages of our approach over the aforementioned ones are twofold. First, our approach accounts for the many complex interactions among prognostic factors, since they could balance each other out. Second, practitioners do not need to make any prior assumption as to which prognostic factor(s) are relevant. Practically, the free and open-source software that we provide allows practitioners to simply list the prognostic factors and behavioural outcome of the intervention, automatically generating questionnaires and allocating participants into groups upon completion of the questionnaires.

Our allocation method uses novel computational tools to infer a participant’s outcome from the prognostic factors, and uses that inferred outcome to allocate the participant. This does not mean that the relationships among prognostic factors and participants’ outcome should be specified before conducting the RCT. Intuitively, imagine that a black box takes as input a participant’s prognostic factors and outputs the participant’s expected outcome. To ensure that groups are comparable at baseline, it suffices to assign participants such that the distribution of expected outcomes among groups are similar. Practically, the black box explicitly structures how the prognostic factors contribute to the outcome. For example, an intervention may aim at improving exercise, with weight as measured outcome. Based on the participants’ age, gender and socio-economic status, the black box simulates how an intervention on exercise may impact their weight. Accordingly, participants are assigned into groups such that the groups have a similar distribution of participant’s simulated impact. These groups might have a different demographic make-up, since they are equalized in terms of their expected trial outcome rather than on prognostic factors. This is more flexible than stratification in small trials (e.g., n < 30) since combinations of prognostic factors may yield the same expected outcome and may thus not have to be kept equal among groups. It is also less simplified than minimization, since it accounts for the interactions of prognostic factors.

We first provide a technical background on the computational solutions to inferring one’s outcome. In particular, we review how participants’ knowledge can be used to deal with vague or conflicting relationships such as those occuring among prognostic factors, and we summarize how the uncertainty of participants’ responses can be incorported into models. Then, we introduce our proposed solution to allocating participants to groups such that groups have a similar distribution of expected response to the intervention. This solution is exemplified via three cases studies, and differences with existing alternatives are also highlighted. We also evaluate our solution in the presence of missing data, for sequential trials, and with the addition of randomness. In order to support practitioners in putting our solution to practice and generate more case studies, we also provide free software that covers all necessary steps, from setting-up an intervention to collecting participants’ questionnaires and allocating them. Finally, we discuss the role that our approach could play in conducting RCTs, and we analyze its current limitations.

## Methods

### Technical solutions to infer behavioural outcomes

Computational approaches typically aim at solving a problem by creating simulations that repeatedly apply a set of rules governing the behaviour of individuals or groups. Therefore, they differ from statistical approaches such as regression models. Computational models of human behaviour have been created in a variety of fields to explain and structure the relationships among prognostic factors and outcome at the individual-level. For example, in criminology, a model of how individuals navigate an urban space was created to explain the locations of crimes (outcome) given offenders’ home locations and the locations of major venues such as shopping malls [8, 9]. Similarly in health psychology, a model of how individuals interact was developed to explain binge drinking (outcome) given demographic information and drinking motives [10]. Numerous methods can explicitly model the mechanisms that link prognostic factors to outcome. In the following paragraph, we will divide these methods as either data-driven or expert-driven. The former (i.e., data-driven) can automatically create a model from the data, but cannot straightforwardly test what an intervention will lead to, since all mechanisms are derived from past data. The latter (i.e., expert-driven) is not automatically built from data but instead involves experts who directly articulate a set of assumptions, which can easily be altered to test what-if scenarios such as the expected consequences if an intervention was to be put to practice.

**Figure 2:**
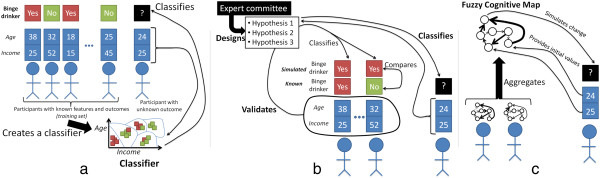
**Computational solutions to infer behavioural outcomes.** The relationships between prognostic factors and the behavioural outcome can be automatically inferred from participants with known prognostic factors and outcomes on entirely mathematical criteria [8] (**a**-classifier obtained from data mining). The relationships can also be theorized by an expert committee and validated on participants with known prognostic factors and outcomes [7] (**b**- man-made model). Our proposed system relies on asking participants about the relationshipsrather than the prognostic factors in isolation (**c**- Fuzzy Cognitive Map).

In data mining, computer algorithms are used to infer the mechanisms (known as patterns) from data where individuals have known prognostic factors and outcome^1^. The result is called a classifier, and it is put to practice on individuals for whom the outcome is unknown (Figure 2a). An example in explaining health behaviour is provided in our recent work on binge drinking, illustrating the added value of classifiers when compared to traditional approaches used in health psychology (e.g., regression analyses) [11]. Conducting a what-if scenario, such as assessing how individuals would behave in the presence of an intervention, would require changing the model (i.e., the structure of the mechanisms) so that it takes into account the new situation. This is difficult when using traditional approaches due to their limitation to linear models, and it is also difficult when using classifiers, because their structure is mathematically defined (e.g., by a set of geometrical cuts in the dataset such as the light blue curves at the bottom of Figure 2a) and it may not be clear how that structure has to change to reflect an intervention. Changing the structure to add an intervention is easier in computational models of health behaviour that are explicitly built from a few (theory-driven) hypotheses specified in advance, such as our model of peer influence involving four hypotheses [10]. The drawback of models built from pre-specified hypotheses is that, unlike classifiers which can be automatically created from data, they require human expertise such as interdisciplinary teams versed in the specific problem (Figure 2b). This lack of a fully automatic procedure to create a model makes it difficult to adapt this approach to new scenarios such as allocating participants in an RCT.

Creating groups with similar expected baseline behavioural outcome in a suitable way for RCTs requires several changes from the aforementioned approaches. First, the procedure must be able to structure the relationships among prognostic factors and behavioural outcome in a fully automatic manner (as for classifiers built using data mining – in line with Figure 2a) since it should be applicable to any trial without requiring human expertise during the randomization procedure. Second, the resulting structure must be easily amenable to changes (as for man-made models – in line with Figure 2b) such that it is possible to assess the expected effect of an intervention. We present an approach that satisfies both requirements (Figure 2c). Its main practical difference compared to the previous two solutions is that all participants must first fill a baseline questionnaire that directly surveys them about the relationships within the prognostic factors themselves and the outcome. This questionnaire can be automatically designed once the prognostic factors and outcome have been specified. Our approach uses neither classifiers nor models based on pre-specified hypotheses: instead, it relies on *Fuzzy Cognitive Maps (FCMs)*, which are artificial intelligence tools used to represent human knowledge. The next section briefly situates FCMs among the techniques used to model human knowledge, and then FCMs are formally specified.

### Modelling human knowledge

Individuals who have managed a condition (e.g., obesity) or performed a certain health behaviour (e.g., smoking) over a long time have gained knowledge about its underlying mechanisms. Historically, individuals have rarely been prompted to directly share this knowledge. Instead, they are more commonly asked about contributing factors in isolation, and associations are inferred statistically. For example, a person may be asked during screening to estimate his/her level of depression and physical activity, and regressions can be used to assess the mechanisms that may link the two factors. However, it is less common to directly ask the person to estimate the impact of depression on physical activity.

This tendency mostly owes to the assumption that individuals cannot accurately reflect on what shapes a condition. This assumption was widely made in older theories, such as the Freudian theory from the late 19th century. Freud proposed a topographical model that separated the mind into conscious, preconscious, and unconscious. Much of the behaviour was supposed to be determined by unconscious thoughts [12], as the mind would actively prevent unconscious traumatic events from reaching consciousness [13]. However, the many psychological systems developed in the last decades have challenged this assumption of behaviours as mostly unconscious, by stating that individuals are to a large extent aware of the mechanisms surrounding their actions. Consequently, theories have been proposed regarding how individuals can reach an unambiguous understanding of such mechanisms [14]. Empirical evidence has also confirmed that individuals can carefully depict how factors influence one another based on complex internal schematics [15]. Several tools have been developed in the last decade, due to the growing importance of patient centered care and the realization that the insight of individuals about the mechanisms can be valuable for behaviour change. In the United Kingdom, a pack of cards named *Agenda Cards* was developed. Each card contains a statement about diabetes and individuals pick cards to indicate which mechanisms and factors are at work for them. These cards have been “very well accepted by people with diabetes and health professionals” [16]. Cards were later developed for obesity in Canada and included statements such as “I do not feel confident in my ability to resist overeating when I am nervous, depressed, or angry” [17].

Participants’ generated-evidence about (causes of) their behaviour has also been used to create models, motivated by the fact that models of complex social phenomena should be built using all evidence available [18]. To build models by prompting participants to share their knowledge, we are asking them to consciously recall facts. This is called declarative memory, and it is part of the long-term memory together with procedural memory (e.g., skills). Within declarative memory, model building taps into the *semantic* memory, which is the conceptualization of the world. That is, participants are not asked to share one specific experience (which may be be representative of their experience as a whole): rather, they are prompted to share the concepts that they have derived from their experiences.

When participants are asked to create models, they might “(un)consciously reduce complexity in order to prevent information overload and to reduce mental effort” [19]. Abundant research has demonstrated that such simplifications occur regardless of expertise [20] or training in model building [19]. Therefore, several approaches have been developed to circumvent this limitation.

The simplest approach is to get the knowledge of a group rather than a single participant. However, experiments have showed that groups show the same biases as individuals and do not result in higher quality decisions [19]. This has motivated the design of mixed methods approaches that use quantitative tools to aggregate the participants’ qualitative answers on questionnaires. In the 1960s, the Delphi method was used to created an aggregate group response by using the median of the individual responses; experiments showed that this leads to more accurate responses than the natural process of group decision-making [21]. In the 1970s, Roberts modelled interactions if they were endorsed by 6 out of 7 respondents, and gave them a directionality if it was agreed to by at least 60% of the respondents [20] pp. 142–179.

Three issues remain in aggregating the knowledge of several participants. First, solving the *conflicts* among participants using a majority vote results in losing some of the individual nuances. Second, relationships vary in weight and capturing this can help focus on the most important drivers of a system. However, the use of linguistic terms to assess the strength of causations (e.g., weak, strong) produces the *vagueness* inherent to human language. Third, there is *uncertainty* in human knowledge and the participants themselves may want to express the extent to which they are confident regarding their knowledge of specific relationships.

FCMs provide the mathematical tools to address these three limitations. FCMs aggregate participants’ experiences using Fuzzy Set Theory (FST), which is precisely “designed to mathematically represent uncertainty and vagueness, and to provide formalized tools for dealing with imprecision in real-world problems” [22]. Here, we are giving particular attention to the two mechanisms through which the uncertainty and vagueness associated with participants’ responses is incorporated in FCMs.

First, participants share their experience via linguistic terms. For example, they may say that weight-based discrimination has had a “strong” impact on their stress level. Perceptions of what constitute a “strong” impact differs among individuals. Rather than associating a term to a specific value, Fuzzy Set Theory associates it to a range (through a *fuzzy membership function*). For example, on a scale from 1 to 5, “strong” may be matched with a normal distribution from 3 to 5 and peaking at 4. Ranges can overlap, which accounts for the possibility that the reality described by a term (e.g., “strong”) partly includes that of another (e.g., “very strong”). The equations of fuzzy membership functions can be found for instance in [8].

Second, the linguistic terms chosen by the participants are aggregated through rules. For example, if 14 out of 42 participants said that a relationship is “strong” while the remaining 28 see it as “very strong” then the first term is associated with a confidence factor of 14/42 while the second is associated with 28/42. The confidence factor represents the uncertainty, and it is central to deriving a numerical value that summarizes the overall experience of the participants. The equations for that derivation are provided for example in [8, 23].

**Figure 3:**
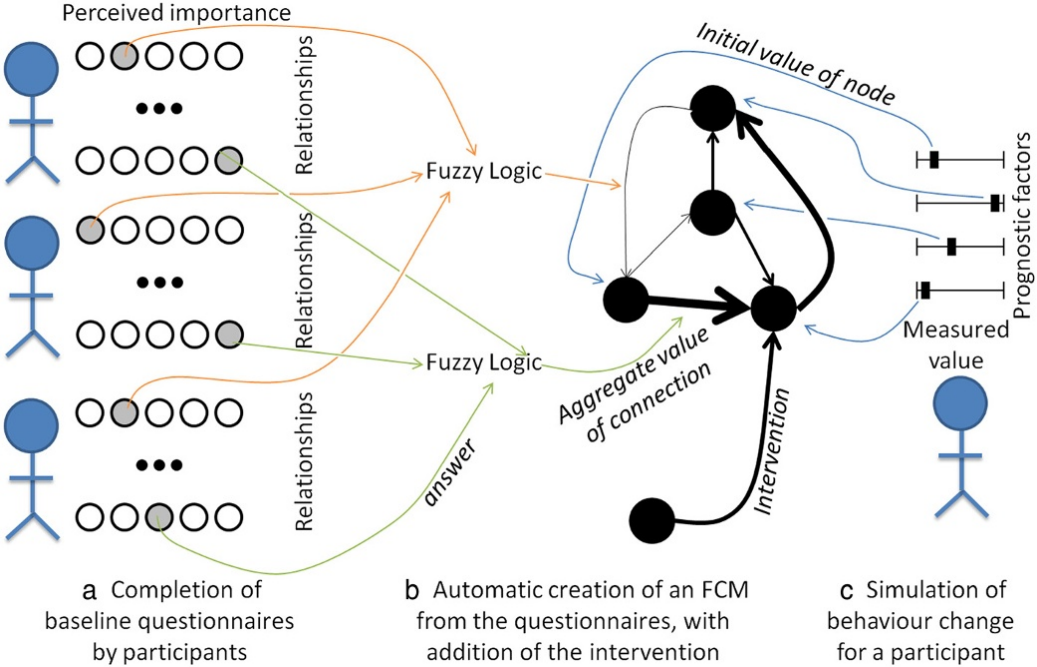
**Construction of a Fuzzy Cognitive Map.** A Fuzzy Cognitive Map (FCM) can be created by asking participants to evaluate the importance of each relationship **(a)**. Each relationship is represented in the map as an edge, whose strength (here depicted by width) is calculated automatically based on participants’ answer **(b)**. A participant’s behaviour is simulated using the values of the participant’s prognostic factors as initial values for the map’s concepts (black circles) **(c)**.

Finally, it should be noted that FCMs differ from several well-known modelling approaches that capitalize on the richness of participants’ experiences. The structure of an FCM can be entirely built from participants’ answers to questionnaires [24–26] (Figure 3a-b). This in contrast to system dynamics, which requires a facilitator to solve the vagueness, uncertainty and conflict found in the perspectives of stakeholders. Bayesian Networks do allow reasoning under uncertainties, but they do not conveniently handle feedback. The latter is essential when structuring the complex relationships shaping behaviour [27].

### The mathematics of fuzzy cognitive maps

Asking participants about their experiences for each relationship and aggregating their answers using FST results in a Fuzzy Cognitive Map. Informally, an FCM uses FST to articulate the relationships among concepts, which can be used to represent prognostic factors. An FCM is graphically depicted as a set of nodes (standing for concepts), linked by directed edges (standing for causality) whose thickness (weight) is computed using FST.

The procedure to develop an FCM is formally described in [28] and applied in a step-by-step manner in [24, 25]; a simplified three-steps version is provided in Figure 3. In this section, we only introduce the mathematics of Fuzzy Cognitive Maps needed to evaluate the validity of the method introduced in this paper. The total number of (prognostic) factors is denoted by *n*. The matrix *W*_*ij*_*, i = 1…n, j = 1…n* denotes the weight of causal relationships from factor *i* to factor *j*, obtained by FST. The initial values for each factor are stored in the vector *V*_*i*_*, i = 1…n*. Each time step of the simulation of an FCM updates the value of all concepts *V*_*i*_ using the following standard equation:


where *f* is a threshold function (also known as *transfer* function) that bounds the output in the range [0, 1]. It is common practice to use such function in order to keep concepts within a specific range [29]. In this work, we use the hyperbolic tanget:


Sigmoid functions such as the hyperbolic tanget are suitable for complex problems [30].

### Proposed solution

In this section, all participants are required to completed a baseline questionnaire on prognostic factors as well as their relationships, and they are then allocated into *n* groups. The procedure abstracted in Table 1 is explained as follows. First, the practitioners give a list of *k* prognostic factors, which are represented as concepts (i.e., nodes) of the FCM (line 1). A concept is then automatically added to represent the intervention (line 2). The edges expressing causality between factors themselves (line 1) as well as between factors and the outcome (line 2) are also generated automatically, but practitioners can eliminate some if they want to incorporate specific assumptions into the trial. This optional elimination can be performed graphically on the computer, as will be illustrated in the discussion.

**Table 1:** **Allocation of all participants to groups after they all provided baseline data**

***Line***	***Action***
1	The FCM has *k* prognostic factors, denoted f_1_, …, f_*k*_ and all linked.
2	The FCM has one intervention linked to the *k* prognostic factors.
3	The Fuzzy Cognitive Map is built from all participants data and denoted FCM(f_1_, …, f_*k*_)
4	For each participant *i*
4-a	The participant’s expected change is computed by E_i_ = FCM(f_1_, …, f_k_)
5	Participants are sorted in ascending order of E_i_
6	Assume that participants must be allocated to *n* groups. For each participant in order:
6-a	Allocate participant i to group (i modulo n)

A questionnaire is automatically generated to ask all participants to evaluate the strength of each possible relationships^3^ (i.e., the weight of each edge) (line 3, Figure 3a). While a regression approach may only include the relationships from prognostic factors to the behavioural outcome, allowing these relationships to run both ways as well as having interactions between prognostic factors is necessary for the presence of loops, which are typically found in complex problems. Upon completion of all the questionnaires, the value of each edge is automatically computed by applying Fuzzy Set Theory on the participants’ answers (Figure 3b). The FCM has then been built.

Once the FCM is built, it is used to infer each participant’s behavioural outcome with and without the intervention (line 4), using each participant’s value for the prognostic factors as an initial value for the corresponding node in the FCM (line 5, Figure 3c). As the FCM inference mechanism has loops in its structure, the values of concepts will change until the behavioural outcome stabilizes. Note that because the behavioural outcome is initially unknown, the FCM is used to assess the relative difference in outcome.

After line 4-a, we have an overall distribution of how much participants would change under the intervention. The goal is then to take equal sizes samples of that distribution such that the samples’ distributions are similar. In other words, we want to allocate participants to each of the groups such that the groups have a similar expected baseline behavioural outcome. To do this, participants are first sorted by their simulated change, from smaller to larger (line 5). Then, the first participant is assigned to the first group, the second to the second group, and so on until all groups received a participant. The allocation then cycles back to the first group, and the cycle repeats until all participants have been allocated (line 6-6a).

## Results

In this section, we contrast the performances of our methods with commonly used alternatives on three case studies. For two of these case studies, we obtained data from human subjects. The data collection was performed in accordance with the Declaration of Helsinki, and it was approved by the ethics committee of Simon Fraser University under the studies 2012 s0725 and 2013 s0494. Informed consent to participate in the study was obtained from all participants. For the last case study, we create a dataset from population-level distributions in order to assess the performance of our proposed methods in the case of large sample sizes.

### Case study 1: designing a RCT for eating disorders

**Figure 4:**
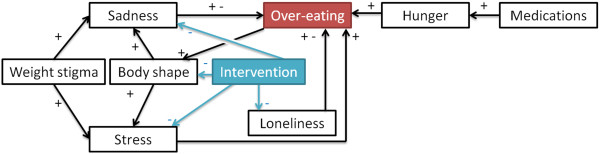
**Design of intervention 1.** Relationships considered for the intervention. A + and a – mean that one concept respectively increases and decreases the value of another. Connections having both + and – offer participants the possibility of choosing increase or decrease.

This case study illustrates a hypothetical intervention aiming to improve participants’ eating patterns by intervening on how emotions (e.g., loneliness, sadness, negative perception of one’s body shape) can translate to food intake. The success of the intervention will be measured by how often participants tend to over eat. Therefore, we want to separate participants into groups such that the groups have a similar distribution at baseline of how participants’ eating patterns would be influenced by the intervention. Assume that the study has taken into account seven prognostic factors and that the intervention would impact four of them as indicated by the blue arrows in Figure 4. In our previous study on the heterogeneity of the drivers of weight [31], 187 young adults (aged 17–28; mean 19.96 ± 1.91) were given questionnaires not only on the prognostic factors but also on their *relationships* in Figure 4. Thus, the relationships’ values in this guiding example are directly drawn from the participants’ answers. The value of the *prognostic factors* was also drawn from data through the same participants regarding sadness, stress, loneliness and medication. Our previous study did not include weight stigma, body shape, over-eating and hunger as part of the questionnaire. As participants provided height and weight, we operationalize body shape as the participant’s Body Mass Index (BMI) category calculated for American adults [32] while weight stigma is set to be directly proportionate to body shape. Due to a paucity of data, the values for both over-eating and hunger are randomly generated from a normal distribution.

**Figure 5:**
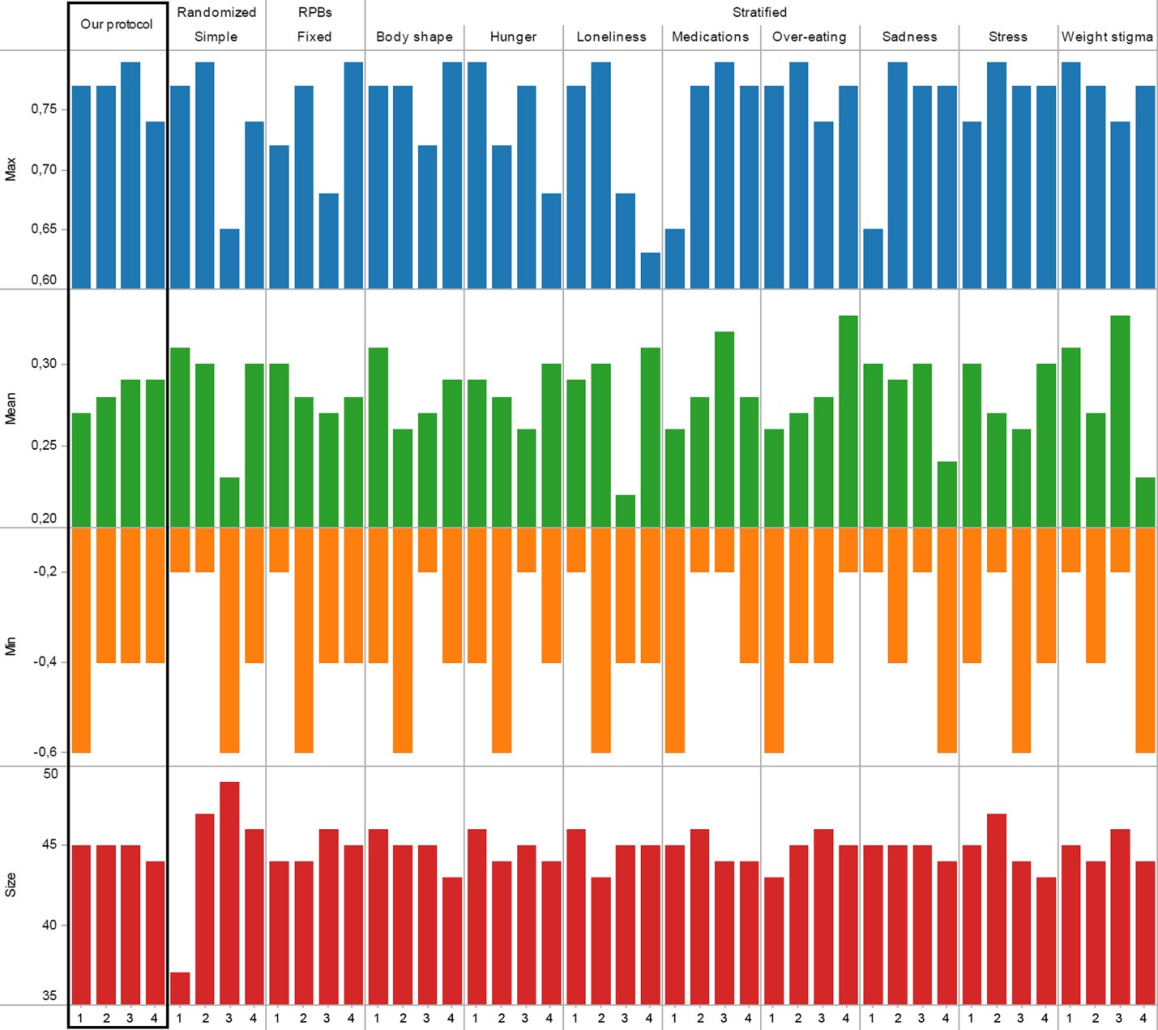
**Results of intervention 1.** Expected behaviour change (absolute min and max, mean) and number of participants (size) when allocating participants into four groups using our protocol, simple randomization, random permuted blocks of fixed size, or stratification.

Given participants’ data and the structure of prognostic factors in Figure 4, the goal was to allocate participants in 4 groups whose changes in over-eating would be similar in reaction to the intervention. Participants were allocated by our protocol and the commonly used techniques surveyed in the introduction (simple randomization, random permuted blocks, stratification using random permuted blocks for each of the 8 prognostic factors). Figure 5 summarizes the expected change in behavioural outcome (min, max, mean) for each of the 4 groups resulting from the 11 allocations. Studies commonly require participants to only report on the prognostic factors and do not ask to evaluate relationships between prognostic factors, possibly because techniques to handle the biases involved (e.g., Fuzzy Set Theory) are not yet widely used. Furthermore, the few studies asking for both factors and relationships may not systematically provide access to individual-level data at baseline and after the intervention, for example because of ethical concerns limiting disclosure to aggregated data. Due to this lack of studies, the behavioural outcome in Figure 5 was computed using a Fuzzy Cognitive Map in lieu of experimental data.

Figure 5 shows that our protocol leads to the most similar groups in terms of size as well as expected behaviour change. Indeed, the standard deviation (the smaller, the more similar the groups) among groups’ mean expected behavioural response is 0.005 for our protocol whereas it ranges from 0.012 (stratification by weight stigma) to 0.038 (stratification by body shape) using other protocols. Similarly, the standard deviation from our protocol is lower than all other protocols on both minimum and maximum expected behavioural response. While this methodology should not be used to provide an indicator for a given protocol in isolation, the notable difference between our suggested protocol and others (e.g., less than half of others’ standard deviation) points to the potential of our approach. These results can be explained as follows. Simple randomization or random permuted blocks both ignore prognostic factors. Thus, in the case of a small RCT (n = 187), there can be significant unbalance in terms of size (simple randomization) or behaviour (both). The performance of the stratification can be affected by the choice of a prognostic factor. For example, using hunger or loneliness results in groups that are very dissimilar on all measures of behaviour change (min, max, mean) whereas prognostic factors such as over-eating or stress result in more balanced groups in term of one measure (max) albeit there are still noticeable differences on other measures (mean, min).

### Case study 2: designing a RCT regarding exercise

**Figure 6:**
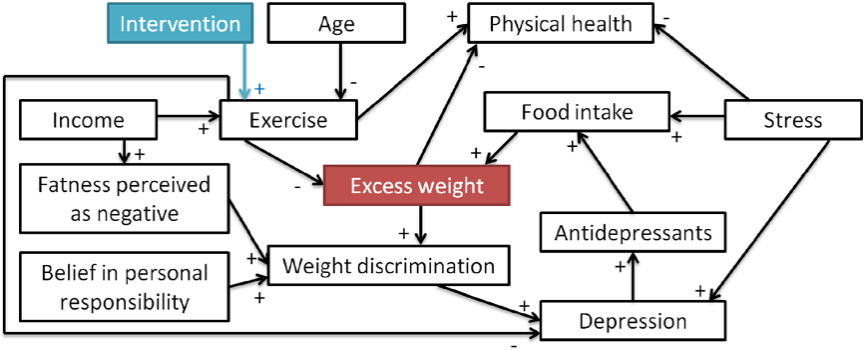
**Design of intervention 2.** Relationships considered for the intervention on exercise.

This case study features an intervention aimed at obesity prevention by improving participants’ exercise. The outcome is measured by excess weight. Therefore, we aim at separating participants into groups whose excess weight is expected to change similarly in reaction to the intervention. The relationships considered in this example are the same as in a previous study on the factors driving adults’ weight [33] (Figure 6). While the previous case study used questionnaires filled by participants in order to provide data on relationships and prognostic factors, this case study will use a simulation to fill questionnaires as if they were answered by actual participants. Synthetic data^2^ allows us to better explore how aspects such as the number of participants affect the results of the different allocation methods.

To ensure that questionnaires are filled in a realistic manner, answers are randomly generated by drawing on real-world data. Note that drawing on real-world data differs from the previous case study: here the answers from participants are generated from a probability distribution calibrated from large samples, whereas in the previous case study each answer was either directly provided by a participant or calculated from what was provided. The age reported is drawn from Statistics Canada [34] using the adult Canadian population as of July 1st 2010, while income uses the 2009 data from Statistics Canada. The answers to five other prognostic factors are also drawn from large Canadian datasets and are separated by age category. We used the National Population Health Survey [35] (NPHS, n = 14,500) for depression and stress, the Canadian Community Health Survey [36, 37] (CCHS) for antidepressants (n = 36,984) and physical health (n = 131,486), and the Prince Edward Island Nutrition Survey [38] (PEINS) for obesity (n = 1,995). The distribution of exercise in the population relies on the observation that most individuals are sedentary [39], which is approximated using an Inverse Gaussian Distribution. An individual’s food intake is set to be slightly above exercise, to account for an environment that promotes eating over exercising. As in the previous study [33], we consider that among Canadian adults, fatness is often perceived as negative and there often is a belief in personal responsibility; thus, both factors are assigned high values. As in the previous case study, we set weight discrimination to linearly depend on obesity. Simulations previously concluded that results obtained by the relationships in Figure 6 were almost indistuinguishable whether weight discrimination was set to depend linearly on weight or when individuals start being discriminated only if very obese [24]. Details of the methodology regarding prognostic factors can be found elsewhere [33]. The strength of the relationships in Figure 6 are drawn from our pilot study in which a panel of participants evaluated each relationship [24]. In other words, the answers of a given virtual participant are set to the ones provided by a previous real participant chosen at random from our panel of respondents. Software to create virtual participants based on the above methodology is distributed with the simulation software.

**Figure 7:**
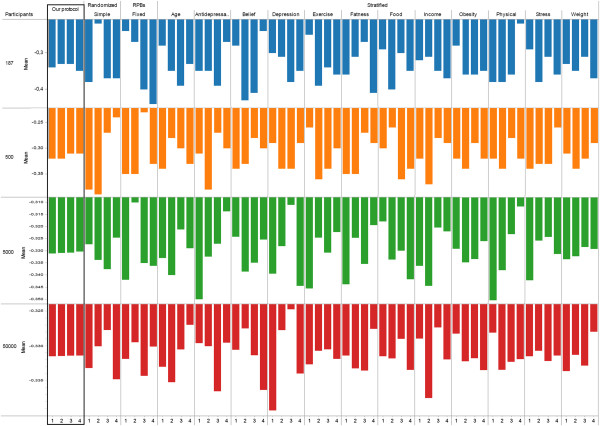
**Results of intervention 2.** Mean expected behaviour change per number of participants and allocation method.

Using the aforementioned methodology, we generated answers to the questionnaires with a sample size of 187 (as in the previous case study), 500, 5000, and 50,000. For each sample, participants were allocated into four groups using all of the methodologies considered in the previous case study (our protocol, simple randomization, random permutation blocks of fixed size, or stratification on one prognostic factor). Our results are summarized in Figure 7. As the sample size increases, the standard deviation on mean expected behaviour decreases, which Figure 7 captures using an increasingly smaller scale. This lowered difference with an increase in sample size is expected due to, for example, “the greater expected balance in proportionate terms between groups” [5]. Nonetheless, our protocol still results in the most similar group in terms of mean expected behaviour, regardless of the sample size considered. For each sample size (n = 187, 500, 5000, 50000) the ratio between the standard deviation on mean expected behaviour of the *best* alternative protocol and our protocol were as follows: 2.69, 3.367, 7.32, 12.92. Furthermore, the capability of our protocol to yield similar groups compared to other protocol increased with the sample size, highlighting that the value of our protocol does not only reside in its use for small trials. The simulation methodology used here does not allow to conclude as to the specific extent of the difference between allocation methods, which would require individual-level data from actual trials. Nonetheless, the observation that groups under our protocol are much more similar compared to other protocols points to the potential of our approach, regardless of sample size.

### Case study 3: designing a RCT regarding unhealthy eating

**Figure 8:**
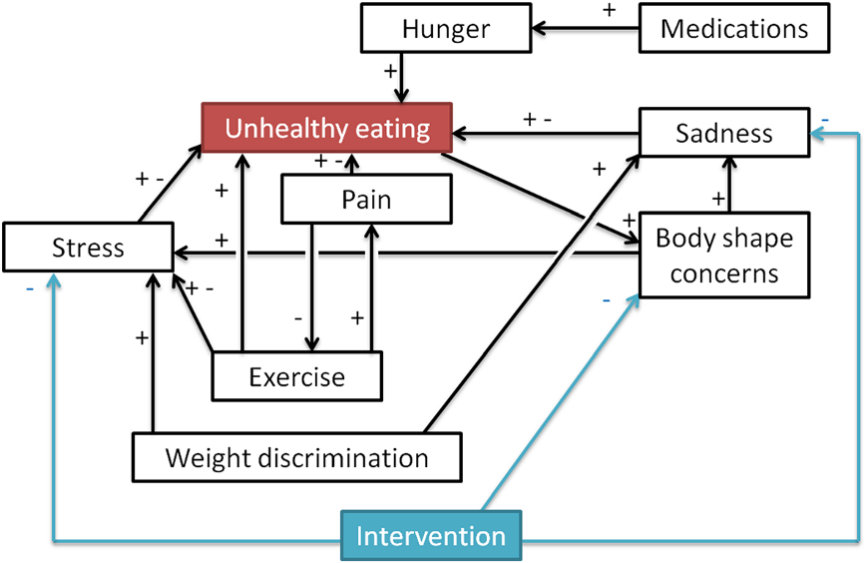
**Design of intervention 3.** Relationships considered for the intervention on unhealthy eating.

This concluding case study features both eating and physical activity behaviours. The objective of the hypothetical intervention is to improve eating behaviour. The relationships considered in this case study are summarized in Figure 8; they are similar to the first case study, with the difference that exercise and pain have replaced loneliness. An online survey was used to collect data from Canadian adults for this case study. Participants were recruited via mailing lists and online posts^4^. A total of 538 participants took part in the study. Participants had the option to only answer the questions that they felt comfortable with. After removing participants who had missing answers, we had a total of n = 430 participants, aged 31.98 ± 12.61, 20.05% male and 79.95% female. All of the relationships featured in this study are populated with the participants’ answers. Sample questions for the relationships featured in Figure 8 are provided in Table 2. Similarly, all of the factors are populated with data from the participants. Therefore, this study did not use any synthetic but only drew on real-world data.

**Figure 9:**
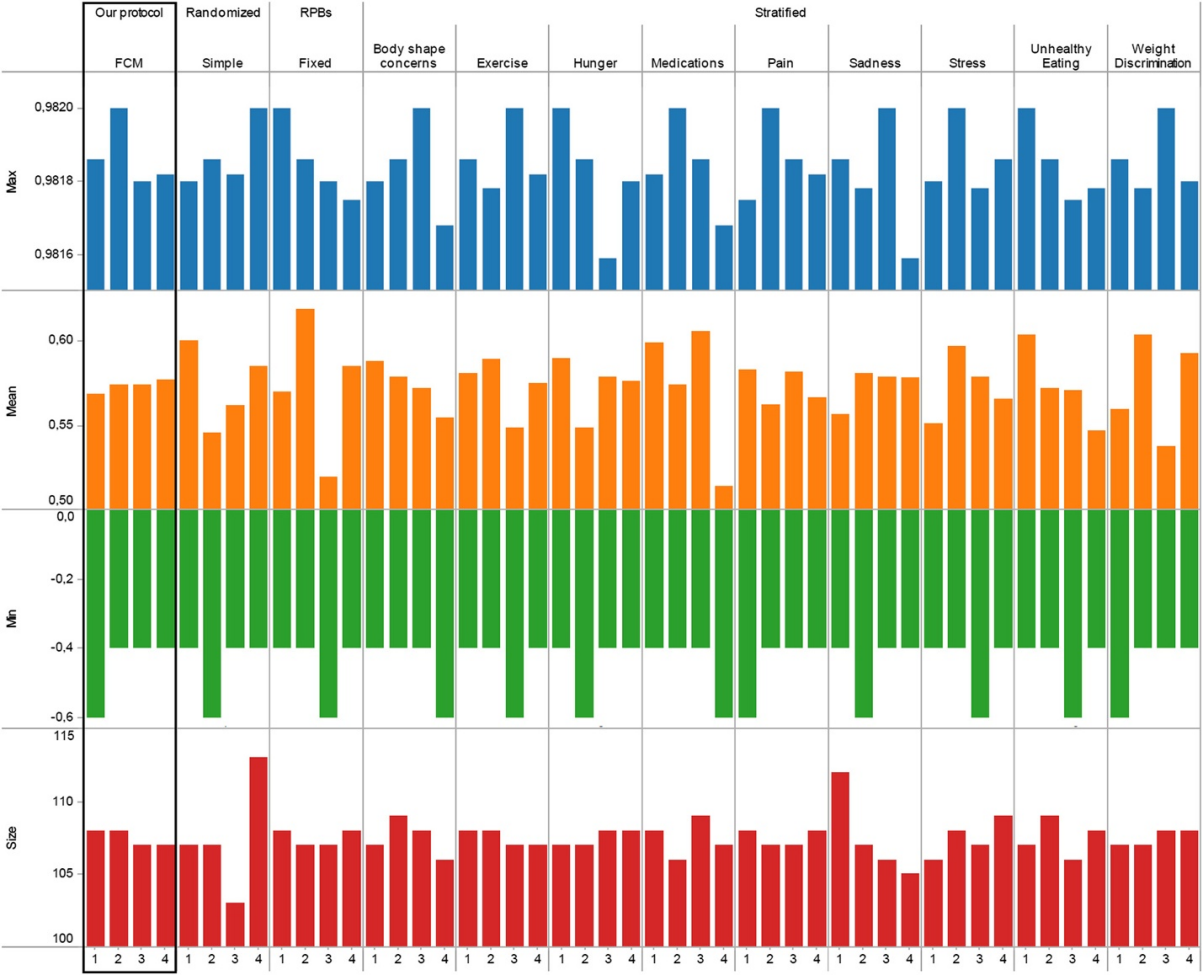
**Results of intervention 3.** Expected behaviour change (absolute min and max, mean) and number of participants (size) when allocating participants into four groups using our protocol, simple randomization, random permuted blocks of fixed size, or stratification.

Figure 9 shows that our protocol leads to the most similar groups in terms of size as well as expected behaviour change. Indeed, the standard deviation obtained from our protocol is one order of magnitude better than most other protocols. This final case study confirms the findings of the previous two case studies regarding the potential of the approach proposed here compared to currently used alternatives.

**Table 2:** **Sample questions used for the relationships depicted in Figure** 8

***Relationship***	***Statements in the online survey (from “never” to “always”)***
Sad →+ Unhealthy eating	When I’m sad, I eat more than I should.
Body shape →+ Stress	I feel stressed because of my body shape.
Pain →- Exercise	When I’m in pain, I avoid exercise.
Exercise →+ Pain	Exercise is painful.
Weight discrimination →+ Stress	Other people’s negative comments or attitudes about my weight make me stressed.
Medications →+ Hunger	I take medications that make me feel hungry.

The full survey can be accessed at: websurvey.sfu.ca/cgi-bin/WebObjects/WebSurvey.woa/wa/survey?142092729.

### Impact of missing data

**Figure 10:**
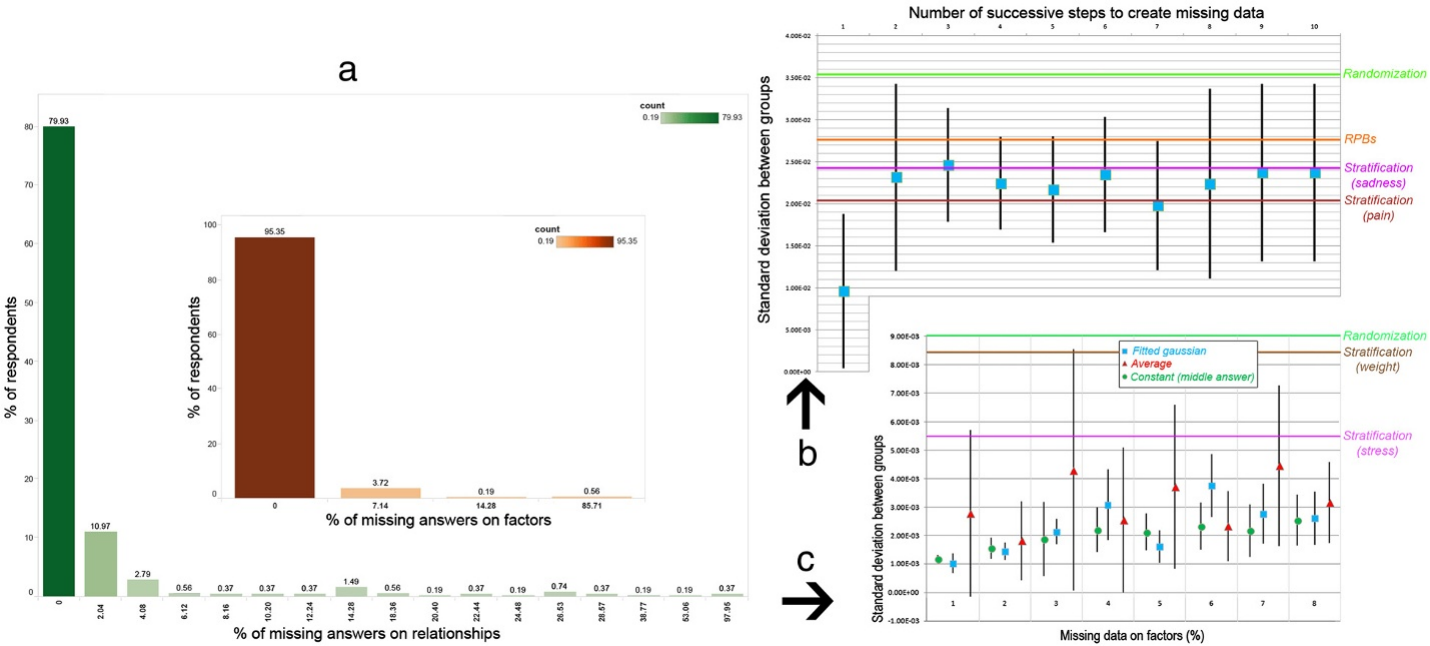
**Impact of missing data.** Distribution of missing data on relationships and factors (inset) in a real-world setting **(a)**. Impact of increasingly missing data on relationships **(b)** and on factors **(c)**; bars represent the standard deviation around the average of multiple runs.

While randomisation methods are often limited in their ability to handle missing data, the scope of data collection required by our method suggests that handling missing data and understanding how it impacts the allocations are important practical considerations. The case study above removed participants with missing answers, resulting in a set of 430 participants. Figure 10a shows the distribution of missing answers among the initial 538 participants, divided by missing answers on either relationships (used to build the FCM) or individual factors (used to provide the individual’s case to the FCM; Figure 10(a) inset). Few answers are missing on individual factors (95.35% of respondents had 0 missing answer) but more are missing on relationships (79.93% of respondents had 0 missing answer). These distributions indicate that our method needs to be robust to a few missing answers on individual factors and to more missing answers on relationships. Consequently, we consider 8% and 20% of missing answers respectively as upper bounds. In order to investigate how the quality of the allocations (measured by the standard deviation between the means of the groups) depends on the percentage of missing answers, we compared the quality of the allocations without missing data to the quality of the allocations obtained when removing a percentage of answers for relationships or prognostic factors.

To remove answers on relationships, we used the previous case study with no missing data (n=430) and transformed answers to “Unsure” with a 5% probability. This was repeated up to 12 times to reach the upper bound on missing relationships. Note that “Unsure” is one of the possible answers in our automatically generated questionnaires, and it is ignored when building the FCM. That is, our method and software already support missing answers in relationships. Results in Figure 10b are reported over 10 runs, with the mean depicted as a square and the standard deviation as a bar. Results show that, as relationships are missing (x-axis), our results are progressively in line with those obtained by stratification (y-axis). Our method can thus operate with a large amount of missing data on relationships and the risk is only to deliver performances similar to the methods most commonly used. Improving our method such that its can operate with up to 20% of missing data while delivering superior performance would be an important goal for future research.

Missing answers on factors are a problem for the methods that allocate participants based on their specific values (e.g., stratification but not randomization). For example, if participants are stratified based on their age category, then a person whose age category was not provided cannot be assigned to a group. Imputation is a typical way to address missing data, by replacing by an estimate. Using imputation, our method can thus operate in the presence of missing data. As for methods such as stratification, the allocation would be affected by the way in which the estimate for missing data is computed. This is illustrated in Figure 10c, where a synthetic population of 5000 individuals was generated using the method of the 2nd case study, and the growing percentage of missing answers (x-axis) was replaced using different imputation strategies (average, fitting a normal distribution, replacing by a constant). The quality of the allocation (y-axis) worsens but remaining better than commonly used methods, for up to 8% of missing data. As noted in [40] p. 280, “missing variables are often from severely ill persons on the skewed end of the distribution” thus nonparametric imputation methods could provide more robust results than regressions or maximum likelihood approaches. We would recommend using a nonparametric method such as the Kernel Density Estimation together with fast evaluation algorithms (e.g., the Fast Gauss Transform [41]) such that missing values can be replaced by good estimates with little computational power even for large trials with numerous factors.

### Fixed sample design versus sequential design

Our proposed method is based on a fixed sample design: the sample size is calculated at the beginning of the trial, all participants complete the baseline questionnaire, and they are then allocated to groups. This is sufficient for many behavioural trials as well as drug trials where immediate randomisation is required. However, many trials fail to reach their planned size within the expected timeline and nonetheless require immediate randomisation. Thus, several sequences are sometimes needed [42] pp. 65–66. Our method can be straightforwardly adapted to work with several waves of recruitments. Assume that a target sample of *n* participants has to be recruited over *s* sequences of allocation with equal sizes, that is, participants are recruited in batches of  and immediately allocated to groups. Instead of applying our method to n participants, it can be applied to them batch by batch. Practically, the FCM can be built on participants 1 to b who are then allocated (1st wave); next, the FCM is built on participants 1 to 2 × *b* (combining questionnaires from the 1st and 2nd waves) who are then allocated, etc. It should be noted that this is an adaptive design, since the ways in which participants in new batches are allocated is based on a data-driven adjustment based on data accumulated so far. This is similar to the covariate-adjusted response-adaptive (CARA) designs investigated by Li-Xin Zhang and van der Laan, in which subjects who join a trial are allocated based on cumulative information from previous subjects, adjusted according to the individual’s information [43, 44].

**Figure 11:**
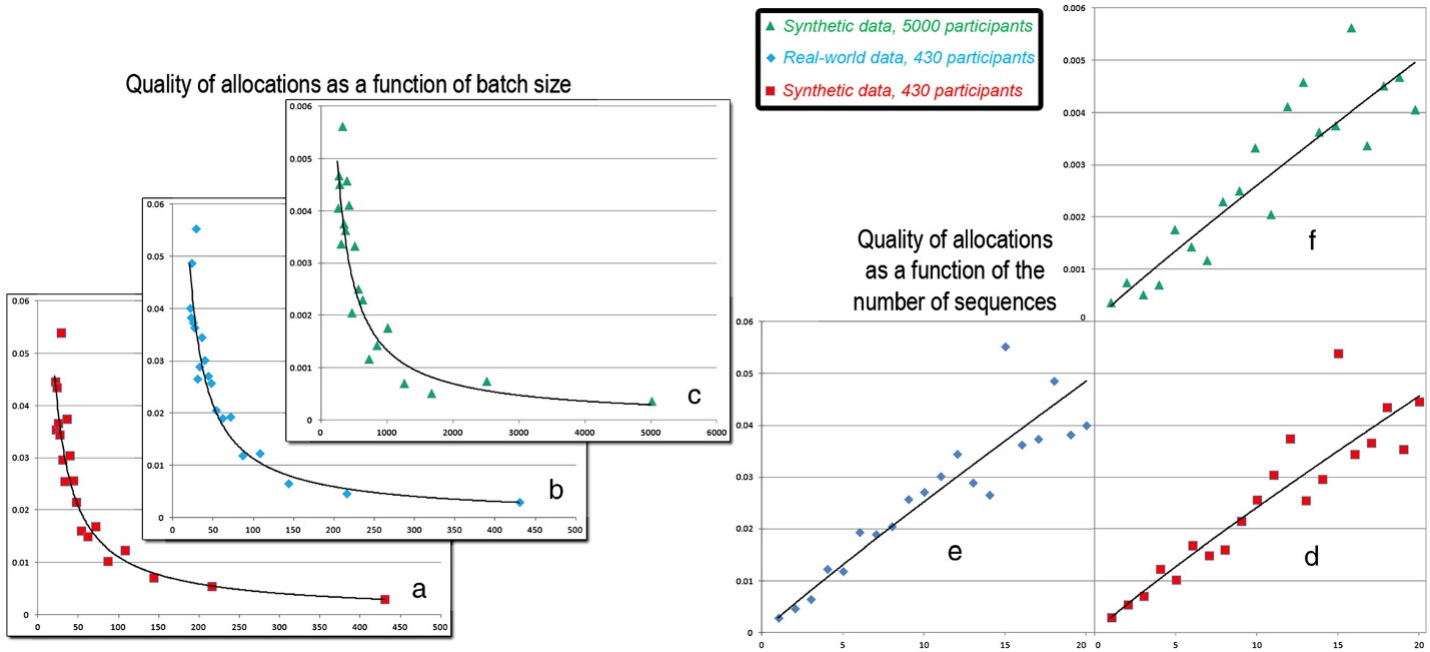
**Impact of sequential allocations.** Quality of the allocations (y-axis) in sequential allocations as a function of the number of participants added at each sequence **(a-c)** or the number of sequences **(d-f)**. Results are similar for synthetic data generated as in the 3rd case study (n=430, n=5000) and real-world data from the 2nd case study (n=430).

The quality of the allocations depends on the desired number of sequences/batch sizes, since the FCM built from a few questionnaires may not be as representative of the underlying dynamics as if it was synthesizing the knowledge of all participants. Figure 11 shows the quality of the allocations (y-axis) as a function of batch’s size (Figure 11a-c) and the number of sequences (Figure 11d-f). The quality of the allocation follows an inverse power law (R^2^ > .85) with the batch’s size, regardless of the size of the desired sample size *n* or whether the data is synthetic/real-world. Consequently, our method may not be adequate if more than 5 sequences are used, but it provides satisfactory results for fewer sequences.

### Impact of randomization

Allocation based on optimization is not randomization since the result is entirely deterministic. The deterministic method that we present still addresses accidental bias as well as selection bias, since the complexity of the protocol carried out by a computer prevents an observer from inferring the group to which a participant would be allocated from the participant’s baseline characteristics. However, randomization provides a basis for the statistical analysis of the trial results [45]. Consequently, optimization-based approaches such as minimization are not randomized in their pure form but often include some randomization in practice. In this section, we show that randomness can straightforwardly be included in our protocol, and we evaluate its impact on the quality of the allocations.

**Figure 12:**
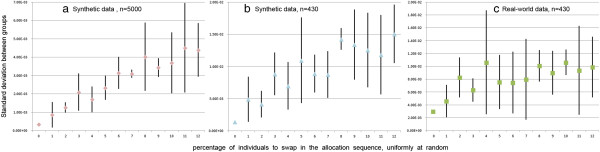
**Impact of randomization.** Quality of the allocations (y-axis) as a function of the proportion of participants who are randomly swapped, using synthetic data generated as in the 3rd case study **(a-b)** and real-world data from the 2nd case study **(c)**.

Our protocol (Table 1) computes the behavioural response of each participant and sorts them by this response. This sequence is then followed to assign individuals to groups, by cycling through groups. For example, assume that the sorted behavioural responses are 1, 3, 5, 6, 8, 9 and that we want 3 groups. We assign the individual having behavioural response 1 to group 1, response 3 to group 2, response 5 to group 3, and then the cycle of group restarts with assigning response 6 to group 1, etc. To introduce randomness, the sequence can be partially shuffled by swapping the positions of some individuals. For example, it could become 1, *6*, 5, *3*, 8, 9. The proportion of individuals who are swapped thus expresses the extent to which the sequence has been randomized. Figure 12 exemplifies that this proportion affects the quality of the allocation. In particular, it shows that datasets generated based on the same rules but with a different number of individuals may be affected differently by randomness (Figure 12a and Figure 12b). In addition, we observe that datasets with the same number of individuals with different underlying patterns are also affected differently by randomness (Figure 12b and Figure 12c). This suggests that, while adding randomness results in less balanced groups, that relationship also depends on both population size and the patterns of individuals’ answers.

While the approach to randomization above is relatively simple to implement, more constrained forms of randomization may be preferrable. Indeed, randomizing the whole sequence may result for example in swapping an individual having a very low expected behavioural response with one having a very high expected behavioural response, which negatively affects the balance of groups. Consequently, swapping individuals that are closer in the allocation sequence may have a lower impact. To favour this, the probability to swap may be made proportional to the distance between individuals in the sequence. If one wishes to fully prevent individuals far apart from swapping, then we would recommend binning the continuous behavioural response estimated by the system. Instead of following the order of participants, the final allocation can thus follow the order of the bins and pick participants within each bin uniformly at random. Such approaches to transforming the data are further discussed in [46], and their consequences on the balance of the groups would have to be investigated in future research.

## Discussion

Bias is a key issue in RCTs, and particularly so for interventions regarding behaviour change. In this paper, we focused on limiting bias coming from allocations, that is, ensuring that participants are allocated to groups which are similar in terms of behavioural response to the intervention. While several methods are readily available, they are typically used to balance prognostic factors in isolation. In other words, the determinants of behaviour are independently balanced, under the assumption that it would lead to balanced behaviours at baseline as well. We took a different approach by focusing on the interactions between prognostic factors via a novel computational method that aims at balancing a simulated behaviour rather than isolated prognostic factors. This method using Fuzzy Cognitive Maps, which have successfully been used in aspects of health where the accuracy of the result could be easily measured and was critical, such as radiotherapy [47] or brain tumor characterization [48]. They have also been used in health behaviour, for example in relation to obesity [24] or diabetes [49].

The potential of our method was assessed through three case studies, using real-world data as well as generated synthetic data from large samples. Results suggest that our method can create groups with more similar behavioural responses than other commonly used approaches. Indeed, other approaches exhibited larger differences between the inferred behaviours of the allocation groups.

To assess the potential of our method, we developed a free and open-source software (CAPT: Computational Allocation of Participants in Trials). Software can be downloaded with no registration needed from http://rctsoft.free.fr, or http://www.crutzen.net/capt, where a detailed tutorial is also provided in order to support practitioners in integrating our solution to the design of their trials and, subsequently, the allocation of participants. The software is stand-alone and has an easy-to-use Graphical User Interface (GUI), so no other programs or writing of syntaxes are needed. Two independent parts are provided in order to provide flexibility to practitioners: the design part and the allocation part.

**Figure 13:**
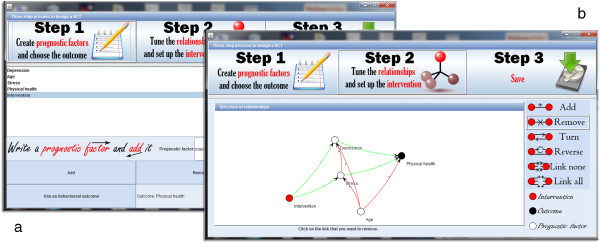
**Design of a trial via our software.** The first step to create a trial is to enter the prognostic factors and pick one as outcome **(a)**. Then, practitioners can state which prognostics factors would be impacted by the intervention **(b)**.

The design part offers a three step process to set-up a trial. First, practitioners list the prognostic factors and pick one as outcome (Figure 13a). Second, they specify what the intervention would impact (i.e., their logic model; Figure 13b). This step also offers the possibility of providing information on how prognostic factors may interact, but practitioners may choose not to impose a structure by continuing with all possible relationships. It is important to stress that ideally, and also for the sake of parsimonity, the relationships provided should be based on the logic model behind the intervention and the prognostic factors that are targeted by the intervention [50]. Finally, the trial is used to automatically generate a questionnaire. Questionnaires can be filled electronically at different study sites (e.g., participants can be recruited in different cities).

**Figure 14:**
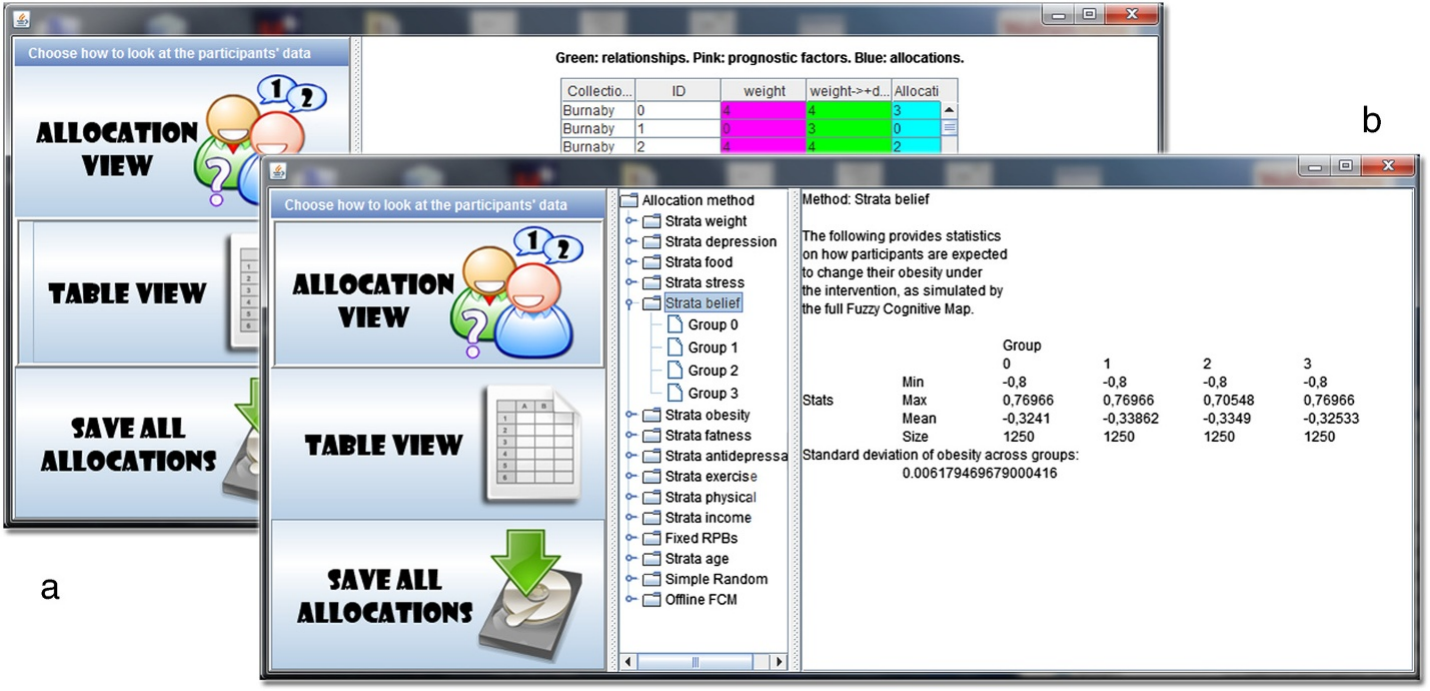
**Assessment of the allocations via our software.** Allocations can be browsed per participant **(a)** or analyzed at different levels **(b)**.

The allocation part will use the completed questionnaires from all available study sites, and allocate participants into the desired number of groups by any of the allocation methods used in the previous section (our protocol, simple randomization, random permuted blocks of fixed size, or stratification on one prognostic factor). The specificities of each participant and his/her allocation can be browsed (Figure 14a) and allocations are organized as a hierarchy in order to be analyzed at different levels (Figure 14b). The root of the hierarchy allows to compare all allocations at once by computing their overlap, which highlights whether allocations tend to allocate participants similarly. Going down the hierarchy by selecting one allocation (Figure 12b) allows to compare all the groups within that allocation in terms of estimated behavioural response. At the deepest level, choosing one group of an allocation provides statistics on the prognostic factors within that group.

Precisely assessing the quality of our approach requires individual-level data in which participants report on relationships and have a known trial outcome. However, because full disclosure is not yet a common practice [51, 52], such data is extremely scarce. One reason is that sharing trial data remains overwhelmingly a matter of choice: for example, less than one out of five authors of a clinical trial(s) were required by their funder to deposit trial data in a repository and, even when asked to do so, only 57% reported doing it [53]. Furthermore, while many studies highlight the benefits of directly asking participants to share their experiences in order to inform the design of interventions [54, 55], they typically focus on *what* drives behaviour (i.e., prognostic factors) rather than *how* (i.e., underlying mechanisms). That is, etiological frameworks do suggest many of the prognostic factors that could be considered when designing a trial, but they rarely operationalize the mechanisms linking prognostic factors to each other and the behavioural outcome. Therefore, the real-world case study used to compare our allocation method to others had to be based on one of the few studies that provided individual-level data and directly asked participants on the perceived impact of prognostic factors [31]. Consequently, we were able to contrast the results of different allocation methods, but individual-level clinical data directly speaking to mechanisms is necessary to adequately assess a given allocation method by itself. In addition, our assessment did not aim to compare our protocol to an exhaustive set of allocation methods. Given the large number of methods available, we focused on the most commonly used ones. A more extensive assessment would have to take into accounts randomization methods such as the maximal procedure [56, 57].

The protocol introduced here can automatically allocate participants into groups once they have provided their completed questionnaires, as illustrated by the free open-source software developed to provide an easy-to-use environment such that practitioners can integrate the protocol to their trials. Two advantages to a fully automatic allocation are as follows. First, there is no risk to obtain sub-optimal allocations by focusing on prognostic factors that turn out not to be as decisive as expected, which can happen when using stratification as exemplified in our case studies. Second, automatically using complex artificial intelligence techniques makes it particularly difficult for staff to know which group a participant will be allocated to, which is a valuable feature given that blinding is difficult trials for behaviour change [1]. One drawback of operating in a fully-automatic mode is that participants are asked to evaluate every possible relationship, and the number of such relationships is proportionate to the square of the number of prognostic factors. When many prognostic factors are considered, operating in a fully-automatic mode can thus contribute to a high quality trial providing the evidence needed to improve healthcare, but at the same time it would increase the burden on participants by generating a larger questionnaire (i.e., not only assessing prognostic factors and outcomes, but also the relationships between them). This may lead an increase in missing data, which our methods can already address but at the expense of lower allocation qualities; this points to the need for further research in handling missing data. The burden placed on participants may also be deemed excessive in some settings. Our software provides a trade-off to practitioners at the trial design stage by allowing them to remove the relationships deemed less relevant for the sake of parsimony, thereby providing input on the logic model behind the intervention [48]. It should be noted that altering the logic model always runs the risk of resulting in a “wrong” model. A model that is “wrong” by assessing inexisting relationships (e.g., impact of age on the weather) would not be a problem in our approach, as participants would be expected to eliminate that relationship. However, a model that is “wrong” by removing important relationships would decrease the quality of the relationships. Based on our simulations for missing data on relationships, we would expect that, as a model increasingly removes important relationships, its performances align with those of stratification.

Future software versions may allow practitioners to directly provide evidence (e.g., from previous studies or literature reviews) regarding select relationships rather than having to systematically query participants. While our method was motivated by trials for behaviour change, enabling practitioners to directly enter equations (e.g., relationships between body tissues [58]) would be an important step toward supporting the application of our method in the clinical setting. Future versions may also offer practitioners the possibility of creating different categories of relationships, which could automatically translate to different phrasing for the questions provided to participants. Many such categories have already been proposed to structure the contributors of behaviour. In Malle’s framework building on folk theory of behavior [59], relationships are classified as reason explanations (e.g., when the environment drives toward an action whose outcome was not desired) or causal explanations (e.g., when the participant has the intention of performing an action that leads to a desired outcome). In the theory of action identification [14], relationships may be seen through a hierarchy where the lowest level dictates simple motor actions (e.g., moving a finger) while the highest level shapes the broader understanding (e.g., the beliefs surrounding a behaviour and its consequences). Categorizing each relationship also offers additional information that can then be leveraged when applying computational methods to infer the participants’ reaction to an intervention. For example, relationships that are higher in the aforementioned hierarchy are also deemed more stables, and they could be attached a larger confidence than relationships regarding lower levels. However, further research is needed to integrate confidence levels into the development of the Fuzzy Cognitive Maps used here. While we developed a method and compared it to commonly used alternatives through multiple case studies, we also believe that the next step of this research should assess how to best guide the analysis of trial data generated by our protocol. Specifically, allocation methods that attempt to improve balance can negatively affect the type I error depending on how the allocation method is accounted for in the analysis. Hagino and colleagues showed that an unadjusted analysis is conservative when stratified randomization or minimization is used, but an analysis adjusting for the allocation factors as covariates does not affect type I error [60]. While we would expect an analysis that ignores our allocation method to be conservative and lose some power, developing specific mechanisms to account for the baseline covariate balancing (resulting from our allocation method) would be critical to “facilitate acceptance of trial results and minimize potential for controversial interpretations” [61].

The protocol that we introduced provided individual-level allocation once all individuals filled the questionnaires, using deterministic artificial intelligence methods. An adaptation was also evaluated to support the sequential enrollment of participants by batches, rather than aiming at reaching the desired sample size before allocating participants. A new version could be developed to satisfy the needs of other possible types of allocations. Specifically, an intervention may be directed at a group rather than a person, or participants may influence each other. In this case, Cluster Randomized Controlled Trials could be used and allocations should take place at the group-level rather than the individual-level. A straigthforward way to using our protocol in this context would be to have all participants still complete the questionnaires, but instead of submitting individual answers to our software, their answers would first be aggregated using Fuzzy Logic to represent the group, and then submitted. However, further simulation research is needed to explore how the aggregation of individual answers into a group answer affects the allocations.

## Conclusion

A new protocol was presented to limit the allocation bias of RCTs, focusing on, but not limited to, interventions on behaviour change. The protocol relies on artificial intelligence techniques that focus on relationships among prognostic factors, thereby providing an alternative to commonly used techniques centred on the prognostic factors themselves.

## Endnotes

^**1**^When an outcome is given, this task is known as *supervised* learning. When there is no outcome, a typical task of *unsupervised* learning is to cluster individuals rather than classifying them, because the outcome is unknown.

^**2**^*Synthetic data* refers to data that was not obtained by direct measurement. In our case, data is generated by a computer in order to observe the reaction of our solution to criteria for which measured data is not available. This process is particularly used to test clinical trials [62] and computational solutions [63].

^**3**^If there are *k* prognostic factors and one behavioural outcome, then the baseline questionnaire has to assess each of the *k*(*k*+1) relationships. This may not be scalable for large *k* due to the resulting size of the questionnaire, and it is possible to remove relationships as summarized in the discussion.

^**4**^A sample invitation to participate is provided in http://web.archive.org/web/20131218220754/ and http://blogs.plos.org/obesitypanacea/2013/12/02/participants-needed-an-online-survey/
